# The glucagon-like peptide-1 receptor agonist exendin-4 ameliorates warfarin-associated hemorrhagic transformation after cerebral ischemia

**DOI:** 10.1186/s12974-016-0661-0

**Published:** 2016-08-26

**Authors:** Fangzhe Chen, Weifeng Wang, Hongyan Ding, Qi Yang, Qiang Dong, Mei Cui

**Affiliations:** 1 Department of Neurology, Huashan Hospital, State Key Laboratory of Medical Neurobiology, Fudan University, No. 12 Middle Wulumuqi Road, Shanghai, 200040 China; 2 The Department of Clinical Laboratory, Central Laboratory, Jing’an District Centre Hospital of Shanghai, Huashan Hospital Fudan University Jing’an Branch, No. 259 Xi Kang Road, Shanghai, 200040 China

**Keywords:** Cerebral ischemia, Exendin-4, Hemorrhagic transformation, Blood-brain barrier, Neuroinflammation, PI3K/Akt-GSK-3β signaling pathway, Warfarin

## Abstract

**Background:**

As the number of patients with cardioembolic ischemic stroke is predicted to be double by 2030, increased burden of warfarin-associated hemorrhagic transformation (HT) after cerebral ischemia is an expected consequence. However, thus far, no effective treatment strategy is available for HT prevention in routine clinical practice. While the glucagon-like peptide-1 receptor (GLP-1R) agonist exendin-4 (Ex-4) is known to protect against oxidative stress and neuronal cell death caused by ischemic brain damage, its effect on preventing warfarin-associated HT after cerebral ischemia is yet unknown. Therefore, we hypothesized that Ex-4 would stabilize the blood-brain barrier (BBB) and suppress neuroinflammation through PI3K-Akt-induced inhibition of glycogen synthase kinase-3β (GSK-3β) after warfarin-associated HT post-cerebral ischemia.

**Methods:**

We used male C57BL/6 mice for all experiments. A 5-mg warfarin sodium tablet was dissolved in animals’ drinking water (effective warfarin uptake 0.04 mg (2 mg/kg) per mouse). The mice were fed for 0, 6, 12, and 24 h with ad libitum access to the treated water. To study the effects of Ex-4, temporary middle cerebral artery occlusion (MCAO) was performed. Then, either Ex-4 (10 mg/kg) or saline was injected through the tail vein, and in the Ex-4 + wortmannin group, PI3K inhibitor wortmannin was intravenously injected, after reperfusion. The infarct volume, neurological deficits, and integrity of the BBB were assessed 72 h post MCAO. One- or two-way ANOVA was used to test the difference between means followed by Newman–Keuls post hoc testing for pair-wise comparison.

**Results:**

We observed that Ex-4 ameliorated warfarin-associated HT and preserved the integrity of the BBB after cerebral ischemia through the PI3K/Akt/GSK-3β pathway. Furthermore, Ex-4 suppressed oxidative DNA damage and lipid peroxidation, attenuated pro-inflammatory cytokine expression levels, and suppressed microglial activation and neutrophil infiltration in warfarin-associated HT post-cerebral ischemia. However, these effects were totally abolished in the mice treated with Ex-4 + the PI3K inhibitor—wortmannin. The PI3K/Akt-GSK-3β signaling pathway appeared to contribute to the protection afforded by Ex-4 in the warfarin-associated HT model.

**Conclusions:**

GLP-1 administration could reduce warfarin-associated HT in mice. This beneficial effect of GLP-1 is associated with attenuating neuroinflammation and BBB disruption by inactivating GSK-3β through the PI3K/Akt pathway.

**Electronic supplementary material:**

The online version of this article (doi:10.1186/s12974-016-0661-0) contains supplementary material, which is available to authorized users.

## Background

Globally, ischemic stroke is one of the leading causes of death and long-term disability [1]. The number of patients with cardioembolic ischemic stroke resulting from nonvalvular atrial fibrillation (AF), the major cause of cardioembolic ischemic stroke, is predicted to double by 2030 [2, 3]. Consequently, a growing burden of warfarin-associated hemorrhagic transformation (HT) after cerebral ischemia can be expected [4–6].

Early HT can occur as a complication of cardioembolic ischemic stroke [7]. Additionally, a higher rate of hematoma expansion and a worse clinical outcome have been reported in warfarin-associated HT patients [8–10]. However, no effective treatment strategy is available for prevention of HT in clinical practice. Experimental studies of cerebral ischemia have established increase in the permeability of the blood-brain barrier (BBB) after ischemia/reperfusion injury as one of the major causes of HT [11, 12].

The glucagon-like peptide-1 receptor (GLP-1R) agonist exendin-4 (Ex-4) is a long-acting analog of the endogenous insulinotropic peptide GLP-1. Both GLP-1 and Ex-4 have multiple physiologic functions, such as the induction of glucose-dependent insulin release, inhibition of glucagon secretion, stimulation of B cell replication, and antiapoptotic action [13]. Owing to their small molecule size, both GLP-1 and Ex-4 can diffuse across the BBB in the central nervous system and provide neuroprotection in cerebral ischemia [14, 15]. While it has been reported that Ex-4 can protect against oxidative products and neuronal cell death caused by ischemic brain damage, it is yet unknown whether Ex-4 is effective in preventing warfarin-associated HT after cerebral ischemia.

Previous studies have shown that after a hemorrhagic stroke, cytotoxic events activate the ubiquitously expressed glycogen synthase kinase-3β (GSK-3β), which increases the expression of β-catenin [16, 17] and subsequently decreases the expressions of claudins [18]. There is substantial evidence that GSK-3β inhibition (tyrosine-216 dephosphorylation) reduces neuronal apoptosis [19–21] and attenuates neuroinflammation in neurodegenerative models [22–24]. Pharmacological stimulation of GLP-1R activates the phosphatidylinositol 3-kinase (PI3K)-Akt signaling pathway, and a number of studies have linked GSK-3β with the PI3K/Akt pathway, thereby showing that phosphorylated Akt inactivates GSK-3β via tyrosine-216 dephosphorylation. Herein, we hypothesized that Ex-4 would stabilize the BBB and suppress neuroinflammation through PI3K-Akt-induced inhibition of GSK-3β after warfarin-associated HT post-cerebral ischemia in mice.

## Methods

### Animals

All experiments were conducted using male C57BL/6 mice (body weight 18–25 g) at a constant temperature and with a consistent light cycle (from 07:00 to 18:00) under normal diet. This study was carried out in accordance with the Guide for the National Science Council of the Republic of China. All animals were treated according to protocols approved by the Institutional Animal Care and Use Committee of Fudan University.

A 5-mg warfarin sodium tablet (Coumadin™, Sigma-Aldrich, St. Louis, MO, USA) was dissolved in 375 mL of water. The C57 BL/6 mice were fed for 0, 6, 12, and 24 hours with ad libitum access to the treated water. Assuming a mouse body weight of 20 g and a water consumption rate of 15 mL/100 g per 24 h, this dosage corresponds to a warfarin uptake of 0.04 mg (2 mg/kg) per mouse over a 24-h period. Similar doses of warfarin have been previously used [25]. After 24 h, the warfarin was withdrawn and middle cerebral artery occlusion was performed (Additional file 1: Figure S1). For the international normalized ratio (INR) measurement, the mice were under deep anesthesia, a peritoneal midline incision was performed, and 0.6 mL blood was drawn from the inferior caval vein as previously described [26]. Blood was transferred to glass tubes (BD Vacutainer^®^) containing sodium citrate as the anticoagulant. Measurements of INR values and prothrombin time were performed in the Department of Central Laboratory, Jingan District Centre Hospital, Shanghai, China.

### Temporary middle cerebral artery occlusion and drug treatment

Mice were anesthetized with ketamine/xylazine (65/6 mg/kg, i.p), and their body temperature was maintained at 37 °C by a heating pad and feedback control system (FHC, Bowdoin, ME, USA). A laser Doppler probe was fixed on the skull 5 mm lateral and 2 mm posterior to the bregma. A coated filament was placed on the right middle cerebral artery (MCA) with concurrent recording of laser Doppler cerebral blood flow to ensure that the cerebral blood flow decreased to below 25 % of the baseline. After 45 min, the filament was removed (Additional file 2: Figure S2). Either Ex-4 (10 mg/kg) or saline was injected through the tail vein immediately after reperfusion. In the Ex-4 + wortmannin group, we intravenously injected 15 μL/kg wortmannin (Sigma-Aldrich), a non-specific, covalent inhibitor of PI3K immediately after reperfusion.

### Assessment of infarct volume, neurological deficits, and blood-brain barrier

All the mice were killed 72 h after temporary middle cerebral artery occlusion (MCAO), and brain tissues were incubated in 2,3,5-triphenyltetrazolium chloride (TTC) for 1 h. The infarct area in each slice was analyzed by a computerized image analysis system, and the infarct volume was calculated by multiplying the distance between sections [27]. Neurological score was determined 72 h after MCAO, according to the graded scoring system described previously by Li et al. [28]. Assessment of motor coordination deficits was performed on days 3 and 7 using the rota rod as previously described [29]. Investigators who performed MCAO models, evaluation of infarct volumes, neurological scales, and rota rod were blinded to all the experimental protocols and drug treatments. To measure BBB permeability, Evans blue (Sigma-Aldrich) was dissolved in saline (2 %) and injected into the right jugular vein 72 h after MCAO. The animals were then killed, and the brain hemispheres were homogenized in 3 mL of *N*,*N*-dimethylformamide (Sigma-Aldrich); incubated for 18 h at 55 °C; and centrifuged. The supernatants were subjected to spectrophotometry at 620 nm.

### Quantification of hemorrhagic transformation

The hemoglobin content in brain tissue was quantified by spectrophotometric assay. The hemispheric brain tissue was homogenized with phosphate-buffered saline (PBS) and centrifuged at 13,000×*g* for 30 min. The hemoglobin-containing supernatant was collected, 80 μL of Drabkin reagent (Sigma) was added to 20-μL supernatant aliquots, and the sample was kept standing for 15 min at room temperature. The optical density in each group was measured at 540 nm, and hemorrhage volume was expressed in equivalent units by comparison with a reference curve generated using homologous blood.

### Western blotting

Striatal brain tissues from the MCA were lysed with radioimmunoprecipitation assay buffer (RIPA) containing protease inhibitors (Sigma-Aldrich, St. Louis, MO, USA). Proteins were separated by SDS-PAGE and then transferred onto a nitrocellulose membrane. The membranes were incubated overnight at 4 °C with the following primary antibodies: anti-p-GSK-3β (Tyr216, 1:1000, Abcam Inc., Cambridge, MA); anti-GSK-3β (1:1000, Abcam); anti-β-actin (1:5000, Sigma-Aldrich); anti-p-β-catenin (Ser33/37/Thr41, 1:2000, Cell Signaling Technology Inc., Danvers, MA); anti-β-catenin (1:1000, Abcam), anti-claudin-3 (1:2000, Santa Cruz, CA); anti-claudin-5 (1:2000, Santa Cruz); anti-p-Akt (Ser473, 1:2000, Cell Signaling); anti-Akt (1:2000, Cell Signaling); anti-ICAM-1 (1:1000, Abcam); anti-VCAM-1 (1:1000, Abcam); anti-IKK-β (1:2000, Santa Cruz); anti-NF-kB (1:2000, Santa Cruz); anti-HHE (1:1,000, Abcam); anti-Iba1 (1:1,000, Abcam); and anti-myeloperoxidase (MPO) (1:2000, Santa Cruz). Secondary antibodies conjugated with horseradish peroxidase were used, and immunoreactivity was visualized by chemiluminescence (SuperSignal Ultra, Pierce, Rockford, IL, USA). Bands of interest were analyzed and quantified using Scion Image.

### siRNA-mediated GSK-3β gene knockdown

The small interfering RNA (siRNA)-mediated GSK-3β gene knockdown was performed as previously described [30]. Briefly, two pairs of GSK-3β siRNAs (21500 R12-1717, R12-1719; Cell Signaling) with a total volume of 4 μL (2 μL each) were stereotaxically injected to the right lateral ventricle following coordinates relative to the bregma: AP = −0.4 mm, L = −1.0 mm, and H = − 2.0 mm (from the brain surface) 48 h prior to MCAO.

### Measurement of cytokine concentration

Striatal brain tissues from the MCA were homogenized and collected by centrifugation at 15,000×*g* for 30 min at 4 °C and then stored at −70 °C until the assay was performed. The supernatant was assayed for tumor necrosis factor-α (TNF-α) and interleukin-1 beta (IL-1β) using enzyme-linked immunosorbent assays (ELISA; R&D Biosystems) as described previously [31].

### Measurement of 8-OHdG formation in the brain

Concentration of 8-hydroxy-2′-deoxyguanosine (8-OHdG) in brain DNA was measured by Piao et al.’s method [32], with slight modifications. Briefly, 200 mg of brain tissue was homogenized in 0.25 M sucrose solution. DNA was extracted from the homogenate under anaerobic conditions. The 8-OHdG content in the brain was measured by using an HPLC-ECD as previously described [33]. Each brain sample was examined in duplicate.

### Immunohistochemistry

Seventy-two hours after MCAO, the mice were anesthetized and first perfused with saline followed by fixation with buffered paraformaldehyde (4 %). The brains were removed and post-fixed in 4 % paraformaldehyde; the paraformaldehyde was then removed and replaced with 30 % sucrose solution overnight. Then 15-μm coronal sections were obtained on a cryostat. The slices were blocked with PBS containing 5 % bovine serum albumin (BSA), 10 % goat serum, and 0.3 % Triton-X 100. Next, the slices were incubated with the primary antibodies anti-Iba1 (1:250, Abcam) and anti-TNF-α (1:100, Santa Cruz) overnight at 4 °C. Then Alexa Fluor 488 or 595 labeled secondary antibody (Molecular Probes Inc., Eugene, OR, USA) for 2 h at room temperature. The tissue sections were washed twice in PBS and then immersed in DAPI (Molecular Probes) solution (1:1000 dilution) for 10 min. The sections were finally rinsed in distilled water and fixed with a coverslip with anti-fade mounting medium.

### Assessment of microglia activation

First, microglia activation were counted and morphologically characterized based on the following criteria. Cells with an oval cell body containing a small volume of cytoplasm and long, thin, delicate, and radially branched processes were classified as ramified microglia [34]. Activated microglia were defined as having an enlarged soma (width greater or equal to 30 μm) and a broad-flattened appearance with the common presence of several lamellapodia [35]. This morphological classification was then confirmed by using a methodology of semi-automatic image analysis to analyze the cell body to cell size ratio in Iba1-stained brain sections as described before [36] by ImageJ software.

### Statistical analysis

All values are expressed as mean ± standard deviation (SD). Differences between means were analyzed using either one-way or two-way ANOVA followed by Newman–Keuls post hoc testing for pair-wise comparison using SigmaStat v 3.5^®^. A *P* value <0.05 was considered statistically significant.

## Results

### Exendin-4 ameliorated warfarin-associated HT after cerebral ischemia

**Fig. 1:**
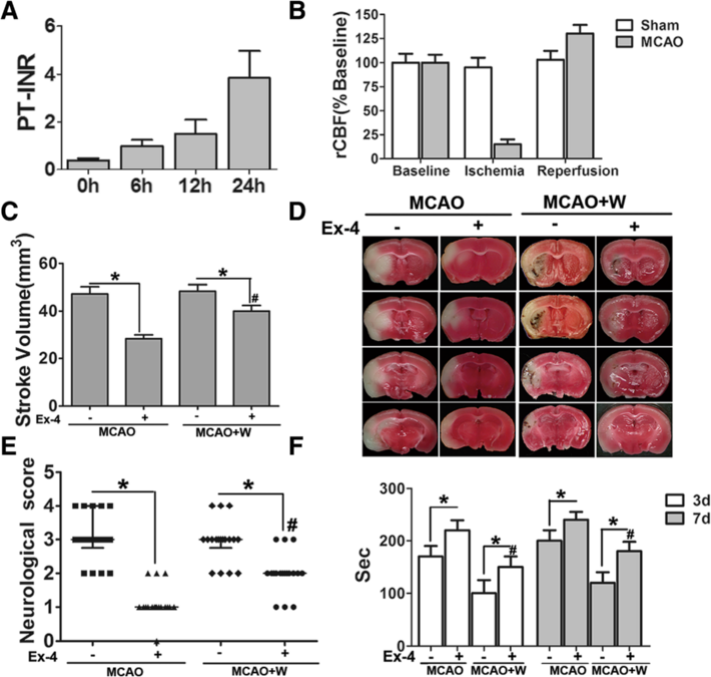
Exendin-4 treatment reduced the stroke volume and improved neurological function after cerebral ischemia. **a** Prothrombin time-international normalized ratio values (PT-INR) in non-MCAO mice after 0, 6, 12, and 24 h of warfarin administration through drinking water. **b** Regional cerebral blood flow (rCBF) in both ischemic and reperfusion stages was recorded using laser Doppler cerebral blood flow. **c** Exendin-4 (Ex-4, 10 mg/kg) was injected through the tail vein immediately after reperfusion. The infarct volume was measured 72 h after middle cerebral artery occlusion (MCAO) using TTC straining. **d** Representative images of TTC straining showing the ischemic area and hemorrhage transformation. **e**, **f** Exendin-4 (Ex-4, 10 mg/kg) was injected through tail vein immediately after reperfusion. The neurological deficits were measured 72 h after MCAO, and assessment of motor function was analyzed on days 3 and 7 using rota rod after MCAO. Data are presented as mean ± SD and analyzed by two-way ANOVA. *P < 0.05 compared with the Ex-4(−) group, ^#^ *P* < 0.05 compared with the MCAO+/Ex-4(+) group

To examine the influence of warfarin on animal PT-INR values, the mice were killed at the indicated time points and the PT-INR values were measured. After warfarin administration, the PT-INR values increased in a time-dependent manner (Fig. 1a). After 24 h of warfarin administration, PT-INR values were elevated (mean = 3.85 ± 1.12; *n* = 6) and reached the therapeutic span used in humans. These results were consistent with those previously reported [26]. In view of these results, we decided to use 24 h as the warfarin administration time for all subsequent experiments.

**Fig. 2:**
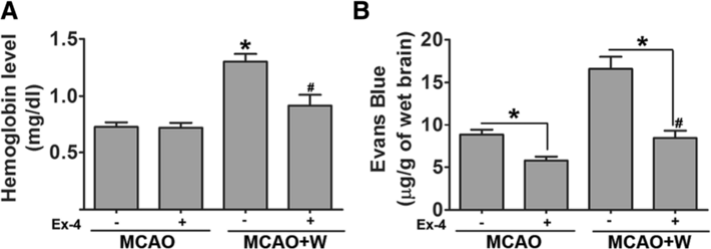
Exendin-4 treatment reduced warfarin-associated HT after cerebral ischemia. **a** Brain hemoglobin levels were evaluated at 72 h after middle cerebral artery occlusion (MCAO). Data are presented as mean ± SD and analyzed by two-way ANOVA. *P < 0.05 compared with the MCAO+/Ex-4(−) group, ^#^ *P* < 0.05 compared with the MCAO+/Ex-4(+) group. **b** Blood-brain barrier (BBB) integrity in MCAO mice were assessed after Evans blue staining. Data are presented as mean ± SD and analyzed by two-way ANOVA. *P < 0.05 compared with the Ex-4(−) group, ^#^ *P* < 0.05 compared with the MCAO+/Ex-4(+) group

MCAO induced a sharp drop of rCBF, leading to extensive infarction in the cerebral cortical and subcortical areas over a series of brain sections in the mice (Fig. 1b, d). Compared with the MCAO+/Ex-4 group, warfarin treatment did not increase the infarct size or neurological deficits. However, warfarin significantly exacerbated HT after cerebral ischemia. Ex-4 suppressed this exacerbation (Fig. 2a). Moreover, Ex-4 showed striking protective effects to reduce to infarct volume and improve neurological function in MCAO mice with or without warfarin treatment (Fig. 1c–f).

### Exendin-4 preserves the BBB integrity in warfarin-associated HT after cerebral ischemia

Functional barrier properties were evaluated using Evans blue assays, 72 h after surgery. Significantly more extravasated dye was measured in the ischemic hemispheres of mice subjected to warfarin treatment compared with the control group. Ex-4 preserved BBB integrity in the model of warfarin-associated HT after MCAO, which was associated with significantly reduced dye extravasation in Ex-4-treated animals (Fig. 2b).

**Fig. 3:**
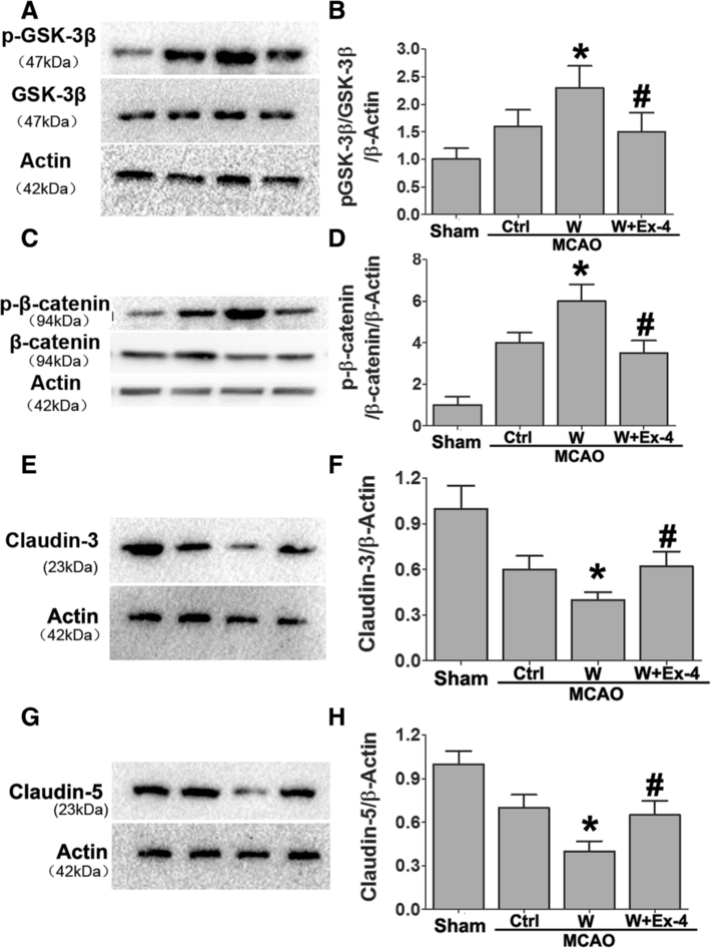
Exendin-4 treatment preserved BBB integrity in warfarin-associated HT after cerebral ischemia. Western blot analysis of **a**, **b** p-GSK-3β/GSK-3β; **c**, **d** p-β-catenin/β-catenin; **e**, **f** claudin-3; and **g**, **h** claudin-5. Representative blots from six independent experiments with similar results are shown. (*Sham*: sham-operated group, *Ctrl*: MCAO group, *W*: warfarin-associated HT group, *W + Ex-4*: warfarin-associated HT pretreatment with Ex-4). Data are presented as mean ± SD from six independent experiments and analyzed by one-way ANOVA. *P < 0.05 vs. MCAO group; ^#^ *P* < 0.05 compared with warfarin-associated HT group

Claudin-3 and claudin-5 are transmembrane proteins essential for maintaining the diffusion barrier provided by tight junctions [37, 38]. Previous studies reported the regulatory role of activation of GSK-3β and β-catenin in claudin-3 and claudin-5 gene expression, respectively [39]. Western blot analyses of the ischemic brain were conducted at 72 h after MCAO. Changes in protein expression of phosphorylated and, therefore, activated GSK-3β (p-GSK-3β, Tyr216) were quantified as a ratio to total GSK-3β (Fig. 3a, b). GSK-3 phosphorylation was significantly increased in the warfarin-treated mice compared with the control group. However, Ex-4 significantly reduced the p-GSK-3β/GSK-3β ratio. Consistent with these results, increased phosphorylated β-catenin levels were also found in warfarin-treated mice compared with the control group (Fig. 3c, d). Ex-4 significantly reduced the p-β-catenin/β-catenin ratio. Tight junction protein expressions were also detected. As shown in Fig. 3e–h, claudin-3 and claudin-5 levels were reduced in the model of warfarin-associated HT after MCAO compared with MCAO mice. However, Ex-4 treatment significantly reversed the reduction.

### Exendin-4 ameliorated warfarin-associated HT after cerebral ischemia through PI3K/Akt/GSK-3β pathway

It has been reported that activated Akt (p-Akt) can inactivate GSK-3β and reduce the amount of GSK-3β available for phosphorylation (through the tyrosine-216 form) [40, 41]. The inactivation of GSK-3β, specifically through tyrosine-216 dephosphorylation, increased β-catenin, which is an important factor in maintaining BBB integrity [42, 43].

**Fig. 4:**
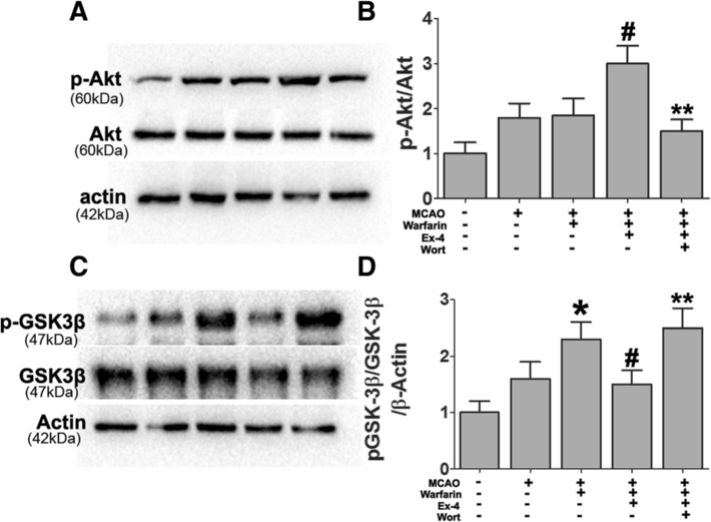
Effect of Ex-4 on PI3K/Akt/GSK-3β pathway in warfarin-associated HT after cerebral ischemia. Mice were intravenously treated either with Ex-4 (Ex-4, 10 mg/kg) or Ex-4 plus the PI3K inhibitor (wortmannin) (15 μL/kg) right after reperfusion. The expression levels of **a**, **b** p-Akt/Akt and **c**, **d** p-GSK-3β/GSK-3β were analyzed by immunoblotting. Representative blots from six independent experiments with similar results are shown. Data are presented as mean ± SD from six independent experiments and analyzed by one-way ANOVA. *P < 0.05 vs. MCAO group; ^#^ *P* < 0.05 vs. MCAO + warfarin group; **P < 0.05 vs. MCAO + warfarin + Ex-4 group

To examine if warfarin and Ex-4 could phosphorylate Akt after cerebral ischemia, the phosphorylation of Akt was examined 72 h after MCAO. After normalizing the values of the active p-Akt with the amount of total Akt (Akt) in each sample, we observed an increase in the Ex-4-treated mice compared to warfarin treatment alone. We indirectly studied the activation of Akt by measuring the phosphorylation of its downstream target GSK-3β in the same brain areas. As compared with the warfarin-treated MCAO mice, Ex-4 treatment significantly suppressed the phosphorylation of GSK-3β. These phosphorylation changes of Akt and GSK-3β were totally abolished when the mice were treated with Ex-4 in combination with the PI3K inhibitor—wortmannin (Fig. 4).

**Fig. 5:**
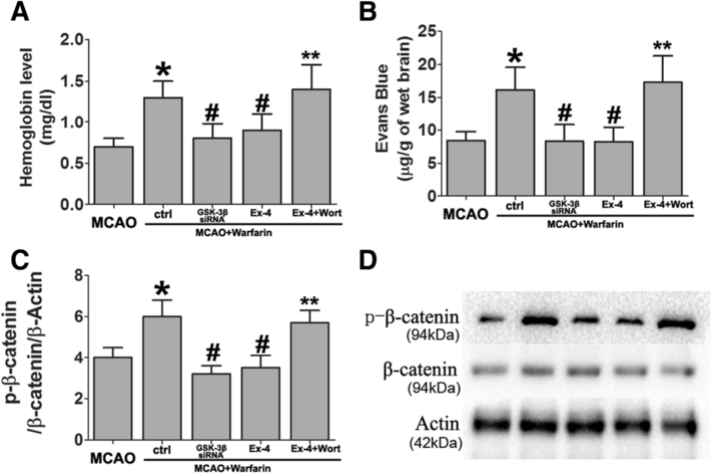
Ex-4 alleviated warfarin-associated HT after cerebral ischemia through PI3K/Akt/GSK-3β pathway. Effects of GSK-3β siRNA and wortmannin on **a** brain hemoglobin level and **b** Evans blue extravasation were evaluated 72 h after MCAO. **c**, **d** The expression levels of p-β-catenin/β-catenin were also been detected by western blotting. Data are presented as mean ± SD from six independent experiments and analyzed by one-way ANOVA. *P < 0.05 vs. MCAO group; ^#^ *P* < 0.05 vs. MCAO + warfarin group; **P < 0.05 vs. MCAO + warfarin + Ex-4 group

These results showed that Ex-4 induced PI3K/Akt pathway activation and subsequent GSK-3β inactivation in the model of warfarin-associated HT after cerebral ischemia. Next, the role of PI3K/Akt/GSK-3β signaling pathway in the integrity of BBB was further investigated using the following antagonists: PI3K inhibitor wortmannin and GSK-3β siRNA in the model of warfarin-associated HT after cerebral ischemia. GSK-3β knockdown by siRNA significantly reduced the warfarin-associated HT. Ex-4 also reversed the warfarin-induced HT in ischemic mice. The mice receiving Ex-4 in combination with wortmannin, however, failed to show this protective effect (Fig. 5a).

### Exendin-4 preserves BBB integrity in warfarin-associated intracerebral hemorrhage after cerebral ischemia through PI3K/Akt/GSK-3β pathway

To detect the role of the PI3K/Akt/GSK-3β signaling pathway in preventing BBB disruption in the Ex-4-treated mice, wortmannin and GSK-3β siRNA were used. In the model of warfarin-associated HT after cerebral ischemia, GSK-3β knockdown by siRNA significantly prevented the warfarin-induced BBB disruption. When administered alone, Ex-4 preserved BBB integrity after warfarin-associated HT. Mice receiving Ex-4 in combination with wortmannin failed to demonstrate reduced dye extravasation into the ischemic brain hemisphere (Fig. 5b).

The effect of GSK-3β siRNA on the expression levels of the p-β-catenin/β-catenin ratio was also measured. GSK-3β knockdown by siRNA significantly reduced the expression of p-β-catenin. As shown in Fig. 5c, Ex-4 reduced GSK-3β activation, thereby stabilizing β-catenin. However, when the mice were treated with Ex-4 and wortmannin, this stabilization effect of Ex-4 was completely lost.

**Fig. 6:**
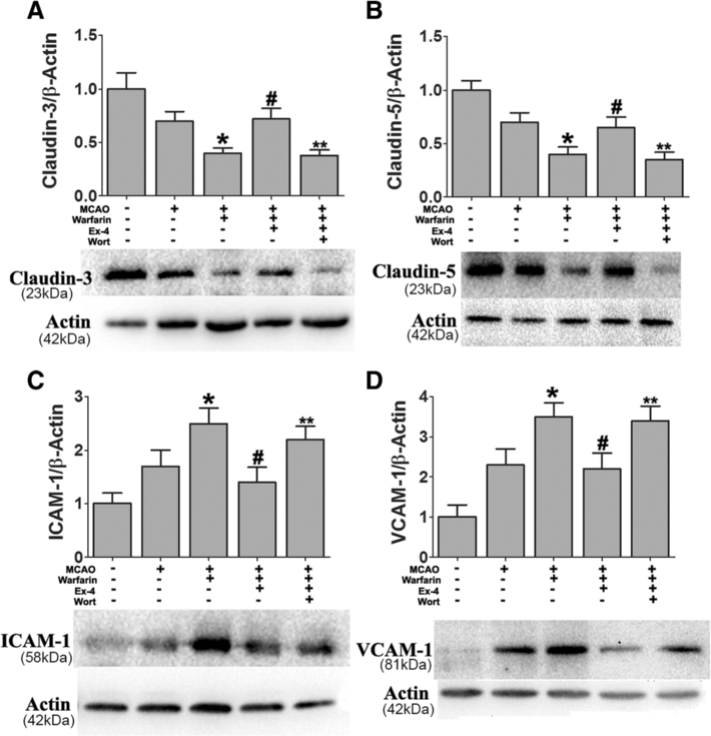
Ex-4 preserved the BBB integrity after warfarin-associated HT through PI3K/Akt/GSK-3β pathway. Mice were intravenously treated with either Ex-4 (Ex-4, 10 mg/kg) or Ex-4 plus wortmannin (15 μL/kg) right after reperfusion. The expression levels of **a** claudin-3, **b** claudin-5, **c** ICAM-1, and **d** VCAM-1 were analyzed by western blotting. Data are presented as mean ± SD from six independent experiments and analyzed by one-way ANOVA. *P < 0.05 vs. MCAO group; ^#^ *P* < 0.05 vs. MCAO + warfarin group; **P < 0.05 vs. MCAO + warfarin + Ex-4 group

The expression levels of tight junction proteins were also detected; warfarin-associated HT reduced claudin-3 and claudin-5 levels. However, Ex-4 treatment significantly increased their expression, and wortmannin reversed the initial increase of claudin-3 and claudin-5 by Ex-4 (Fig. 6a, b).

The PI3K/Akt pathway has been implicated in stabilization of the BBB through decreased expression of endothelial adherent proteins vascular cell adhesion molecule-1 (VCAM-1) and interstitial cell adhesion molecule-1 (ICAM-1) [44, 45]. Warfarin-associated HT significantly increased the expression of ICAM-1 and VCAM-1. Both adhesion molecules’ expressions were decreased by Ex-4 treatment, and wortmannin reversed the reduction of the adhesion molecules’ levels induced by Ex-4 (Fig. 6c, d).

### Exendin-4 suppresses oxidative DNA damage and lipid peroxidation in warfarin-associated HT after cerebral ischemia

**Fig. 7:**
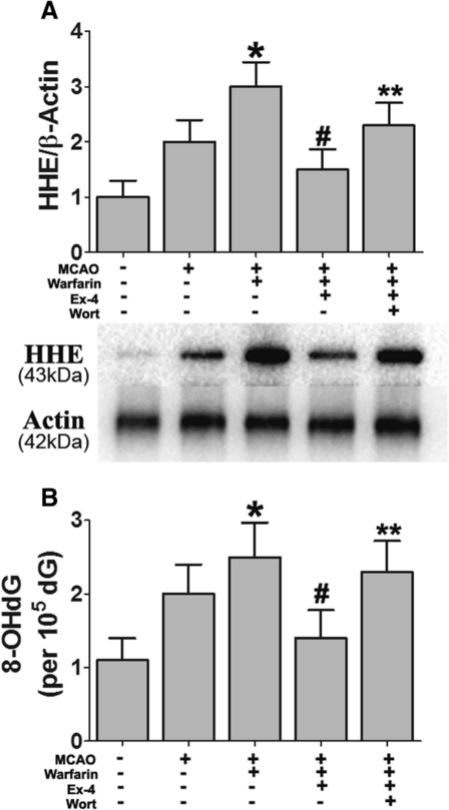
Ex-4 suppressed oxidative DNA damage and lipid peroxidation in warfarin-associated HT after cerebral ischemia. Mice were intravenously treated with either Ex-4 (Ex-4, 10 mg/kg) or Ex-4 plus wortmannin (15 μL/kg) right after reperfusion. The expression levels of **a** HHE and **b** 8-OHdG in the brain tissue were detected. Data are presented as mean ± SD from six independent experiments and analyzed by one-way ANOVA. *P < 0.05 vs. MCAO group; ^#^ *P* < 0.05 vs. MCAO + warfarin group; **P < 0.05 vs. MCAO + warfarin + Ex-4 group

Next, we investigated whether Ex-4 can control oxidative stress in warfarin-associated HT using lipid peroxidation indicator (HHE) and DNA oxidative injure indicator (8-OHdG). 8-OHdG is a major form of oxidative DNA damage product, and 4-hydroxyhexenal (HHE) is one of the major lipid peroxidation products that are formed by n-3 polyunsaturated fatty acids in cells exposed to oxidative stress [46]. The expression levels of 8-OHdG and HHE were significantly increased in warfarin-associated HT brains compared to MCAO brains. The levels of these oxidative stress markers were significantly decreased in the Ex-4-treated group. When the mice were treated in combination with wortmannin, Ex-4 failed to suppress the expression levels of 8-OHdG and HHE (Fig. 7).

### Exendin-4 attenuated pro-inflammatory cytokines in warfarin-associated HT after cerebral ischemia

**Fig. 8:**
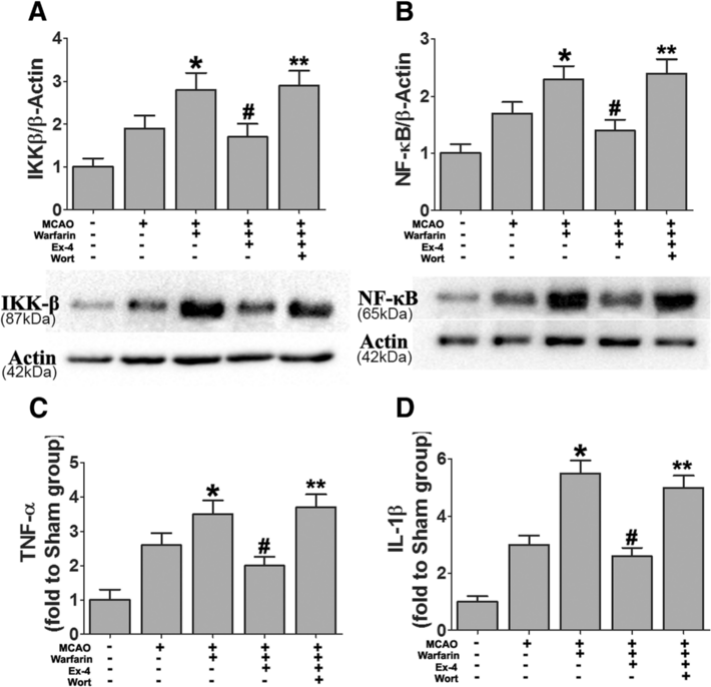
Ex-4 attenuated pro-inflammatory cytokines in warfarin-associated HT after cerebral ischemia. Mice were intravenously treated with either Ex-4 (Ex-4, 10 mg/kg) or Ex-4 plus wortmannin (15 μL/kg) right after reperfusion. The expression levels of **a** IKK-β and **b** NF-kB were analyzed by western blotting. The concentrations of pro-inflammatory cytokines **c** TNF-α and **d** IL-1β were detected by ELISA. Data are mean ± SD from six independent experiments and analyzed by one-way ANOVA. *P < 0.05 vs. MCAO group; ^#^ *P* < 0.05 vs. MCAO + warfarin group; **P < 0.05 vs. MCAO + warfarin + Ex-4 group

We additionally examined the role of Ex-4 in modulating neuroinflammation by measuring expression levels of several cytokines such as IKK-β, NF-kB, TNF-α, and IL-1β. The expression levels of IKK-β and NF-kB were significantly increased after warfarin-associated HT compared to MCAO alone, while Ex-4 treatment reduced the effect and wortmannin blocked the reduction induced by Ex-4 (Fig. 8a, b).

The expression levels of TNF-α and IL-1β were evaluated by ELISA. Both these cytokines were upregulated in warfarin-associated HT mice, and Ex-4 blocked the increase concordantly with a similar pattern for IKK-β and NF-kB. The modulating effect of Ex-4 on the cytokines’ expression levels were reversed by co-treatment with wortmannin (Fig. 8c, d).

### Exendin-4 suppresses neuroinflammation in warfarin-associated HT after cerebral ischemia

**Fig. 9:**
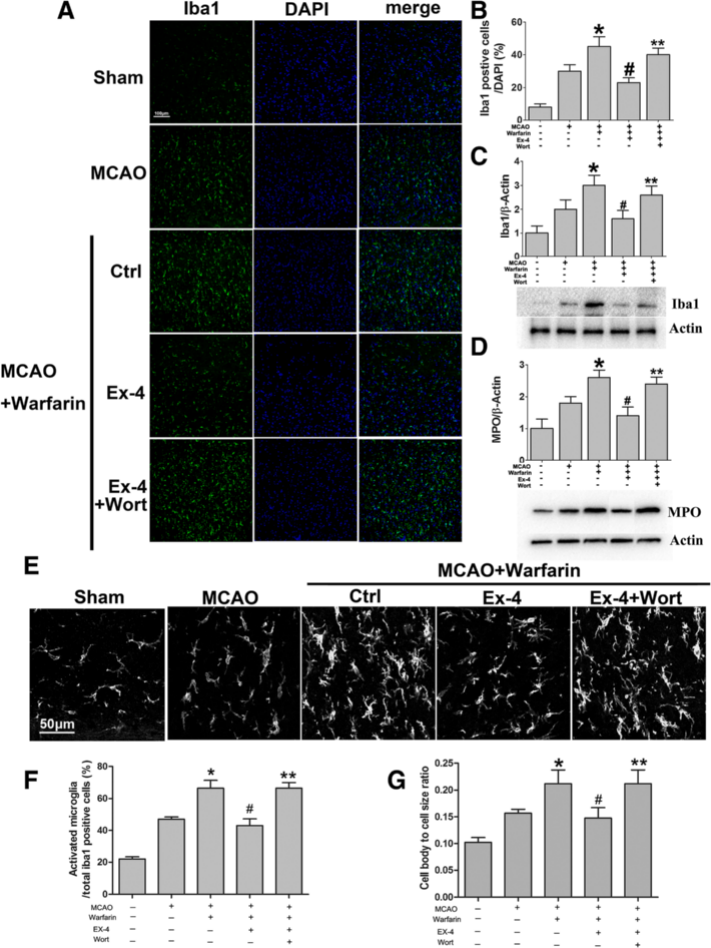
Ex-4 reduced Iba1+ microglial/macrophage cells and neutrophil infiltration in warfarin-associated HT after cerebral ischemia. Immunostaining of Iba1 was performed in the cortical and subcortical areas supplied by the middle cerebral artery. **a** Representative immunofluorescence images showed Iba1+ (*green*) and DAPI+ (*blue*) microglia/macrophages in the Ex-4-treated mice compared to the warfarin-associated HT group. *Scale bar* 100 μm. **b** Quantitative analysis of Iba1+ cells. The expression levels of Iba1 (**c**) and MPO (**d**) were detected by western blotting. **e** Representative images show microglial morphology in different groups. *Scale bar* 50 μm. **f** The number of activated microglia was expressed as a percentage of the total number of Iba1+ cells. **g** The cell body to cell size ratio of microglia provides additional information about microglial activation. Data are presented as mean ± SD from six independent experiments and analyzed by one-way ANOVA. *P < 0.05 vs. MCAO group; ^#^ *P* < 0.05 vs. MCAO + warfarin group; **P < 0.05 vs. MCAO + warfarin + Ex-4 group

Consistent with the changes in cytokine levels, immunofluorescence analysis also showed that warfarin-associated HT robustly enhanced immunofluorescence intensity of Iba1 staining (a marker of microglia/macrophages) in the MCA area compared to the MCAO group (Fig. 9a). The quantification results showed Iba1-positive cells were significantly attenuated in the mice treated with the Ex-4 (Fig. 9b). Further, morphology analysis showed that the number of activated microglia was attenuated in the Ex-4-treated group (Fig. 9e–g). Consistent with these results, double immunofluorescent staining showed Iba1+/TNF-α + cells were elevated in the warfarin-associated HT group and Ex-4 significantly reduced the double positive cells (Additional file 3: Figure S3). Wortmannin blocked this function of Ex-4. Western blotting showed similar results with immunostaining (Fig. 9c). Taken together, these results suggest that the protection conferred by Ex-4 was likely mediated by the inhibition on warfarin-associated neuroinflammation after cerebral ischemia.

In addition to brain resident microglia, hematogenous leukocytes have been shown to play a pivotal role in post-stroke neuroinflammation. Among white blood cells, neutrophils have attracted much interest recently and have been intensively studied. The level of myeloperoxidase (MPO) was significantly increased in the warfarin-associated HT group compared to MCAO. Ex-4 treatment reversed the MPO level. The inhibition of Akt by wortmannin restored the MPO level back to that of the warfarin-associated HT group (Fig. 9d).

## Discussion

Atrial fibrillation is a severe independent risk factor of stroke, its attributable risk increasing with age up to more than 20 % [47]. INR-driven oral anticoagulation with vitamin K antagonists to an INR of 2–3 reduces the risk of an ischemic stroke by over 60 % and has been the standard of stroke prevention in patients with AF [48]. However, anticoagulation therapy is closely related to HT after ischemia. In addition, cardioembolic stroke also carries with it an increased risk of HT [49]. The chief mechanism of HT is considered to be blood leakage due to disruption of the BBB. Our results showed that pretreatment with warfarin could significantly increase the INR level in a time-dependent manner and dramatically enhance Evans blue leakage provoked by MCAO. Although the infarct volume and neurological deficits were not significantly different between the groups with or without warfarin treatment, warfarin significantly promoted the HT after cerebral ischemia, which is consistent with the permeability measurement results.

GLP-1 and long-acting Ex-4 induce numerous biological actions through the G protein-coupled GLP-1 receptor (GLP-1R). GLP-1R is reportedly expressed in a wide range of tissues, including the brain. Moreover, GLP-1R stimulation has shown neuroprotective actions in previous findings, thereby establishing that GLP-1R stimulation protects hippocampal neurons from amyloid-β peptide and glutamate-induced toxicity [50, 51]. As the GLP-1R agonist Ex-4 is permeable to the BBB with a relatively long half time, it has possible clinical applications. Several studies have shown that Ex-4 can protect against oxidative products and neuronal cell death caused by ischemic brain damage [15]. However, to the best of our knowledge, whether GLP-1R stimulation is associated with warfarin-associated HT has not yet been studied. Herein, we reported that Ex-4 prevented the exacerbation of HT caused by warfarin without affecting the infarct volume. The mechanism whereby Ex-4 prevented the exacerbation of HT might involve maintenance of the expression of tight junction proteins and suppress the neuroinflammation associated with warfarin treatment. The pathways that strengthen the antiapoptotic and neuroprotective effects of Ex-4 after cerebral ischemia mostly converge on activation of the transcription factor cAMP response element-binding protein (CREB) by phosphorylation. In the present study, the PI3K/Akt-GSK-3β signaling pathway appeared to contribute to the protection afforded by Ex-4 in the warfarin-associated HT model.

PI3K/Akt plays a crucial role in the cell death/survival pathway through several different downstream targets including GSK-3β [52]. A temporal increase in phospho-Akt after cerebral ischemia has been reported, and GSK-3β dephosphorylation at tyrosine-216 is accelerated as a downstream target of Akt [53]. The inactivation of GSK-3β via tyrosine-216 dephosphorylation mediates neuronal survival after cerebral ischemia [43]. In addition, the inactivation of GSK-3β results in stabilization of β-catenin, a protein that plays a role in cell adhesion. As a result, free β-catenin is allowed to accumulate and be translocated to the nucleus, binding to the transcription factors to alter target gene expressions [54], such as those of tight junction proteins claudin-3 and claudin-5 [18, 39]. Furthermore, GSK-3β inactivation may also decrease NF-kB expression, thereby reducing neuroinflammation.

In this study, Akt phosphorylation at Ser473 and GSK-3β dephosphorylation at tyr216 were increased in warfarin-associated HT after cerebral ischemia. Administration of Ex-4 substantially decreased HT and maintained the stability of BBB. The reduced dye extravasation and brain hemoglobin level were similar to that achieved by inhibition of GSK-3β. Evidence supporting enhanced BBB stabilization by Ex-4 including decreased adherens (VCAM-1 and ICAM-1) and increased tight junction (claudin-3 and claudin-5) proteins could be totally abolished by wortmannin, a specific PI3K inhibitor. These results suggest that warfarin-associated HT reduced the expression of tight junction proteins. This effect was prevented by treatment with Ex-4 through the PI3K/Akt-GSK-3β pathway. Furthermore, Ex-4 reduced the warfarin-induced hemorrhage volume via a protective effect on vascular endothelial cells.

Inflammation has been recognized as a key contributor to the pathophysiology of cerebral ischemia [55]. Inflammation includes a series of cellular events such as infiltration of neutrophil cells and activation of microglia/macrophages and astrocytes [56]. We found that warfarin-associated HT significantly upregulated Iba1-positive cells. Microglia/macrophage activation, together with elevated expression of pro-inflammatory cytokines such as IKK-β, NF-kB, TNF-α, and IL-1β, demonstrated that the warfarin-associated HT induced a neuroinflammation after cerebral ischemia. It has also been reported that activated microglia/macrophages are major sources of metalloproteinase generation, which is closely associated with ischemia-induced cerebral hemorrhage and edema. NF-kB is a central mediator of these inflammatory processes. Recent evidence has shown that the PI3K/Akt signaling pathway may be an endogenous negative feedback regulator of NF-kB-mediated pro-inflammatory responses [57, 58]. Several pro-inflammatory NF-kB target genes including TNF-α and IL-1β could mediate the deleterious effects on neurons under ischemic conditions. In the present study, we showed that warfarin-induced HT markedly induced the activation of microglia/macrophages and consequently increased the production of pro-inflammatory cytokines and Ex-4 significantly inhibited the neuroinflammation induced by warfarin through the PI3K/Akt-GSK-3β pathway. Moreover, suppression of oxidative damage is also a key factor in neuroprotection. Using 8-OHdG and HHE as markers of oxidative stress, our study showed that Ex-4 reduced the warfarin-induced accumulation of oxidative DNA damage and lipid peroxidation after cerebral ischemia.

## Conclusions

Our study results showed that administration of GLP-1 could reduce warfarin-associated HT in mice. This beneficial effect of GLP-1 was associated with attenuating neuroinflammation and BBB disruption by inactivating GSK-3β through the PI3K/Akt pathway. These findings have important clinical implications and would be particularly beneficial in those receiving anticoagulant therapy. Future clinical trials should focus on confirming the efficacy and safety of this therapy.

## Additional files

Additional file 1: Figure S1. The PT-INR values after warfarin withdrawal. After warfarin withdrawal, INR values remained stable for the next 6 h and dropped to normal values after 24 h. Data are shown as mean ± SD. Additional file 2: Figure S2. The rCBF levels in the ischemia and reperfusion stages in MCAO mice. A coated filament was placed on the right middle cerebral artery (MCA) with concurrent recording of laser Doppler cerebral blood flow. In the ischemia stage, the rCBF decreased to <25 % of baseline. After 45 min, the filament was removed and the rCBF increased to 110 % of baseline. Additional file 3: Figure S3. Representative immunofluorescence images showed co-localization of Iba1 (green) and TNF-α (red) in microglia. Immunostaining of Iba1(green), TNF-α(red), and DAPI (blue) was performed in the cortical and subcortical areas supplied by the middle cerebral artery. (**A**) Representative immunofluorescence images showed the percentage of Iba1+/TNF-α + cells to total Iba1+ cells was increased after warfarin treatment. EX-4 treatment reduced the Iba1+/TNF-α + cells percentage, whereas wortmannin blocked this effect of EX-4. Scale bar 50 μm. (**B**) Quantitative analysis of Iba1 and TNF-α double positive cells/Iba1-positive cells.

## Abbreviations

8-OHdG: 8-hydroxy-2′-deoxyguanosine
AF: Atrial fibrillation
BBB: Blood-brain barrier
Ex-4: Exendin-4
GLP-1R: Glucagon-like peptide-1 receptor
GSK-3β: Glycogen synthase kinase-3β
HHE: 4-hydroxyhexenal
HT: Hemorrhagic transformation
ICAM-1: Interstitial cell adhesion molecule-1
IL-1β: Interleukin-1 beta
INR: International normalized ratio
MCAO: Middle cerebral artery occlusion
PBS: Phosphate-buffered saline
PI3K: Phosphatidylinositol 3- kinase
RIPA: Radioimmunoprecipitation assay buffer
TNF-α: Tumor necrosis factor-α
TTC: 2,3,5-triphenyltetrazolium chloride
VCAM-1: Vascular cell adhesion molecule-1
