# Supplementary figures and images for: The glucagon-like peptide-1 receptor agonist exendin-4 ameliorates warfarin-associated hemorrhagic transformation after cerebral ischemia
Source: J Neuroinflammation. 2016 Aug 26;13(1):204. doi: 10.1186/s12974-016-0661-0 (PMC5002167; doi:10.1186/s12974-016-0661-0)

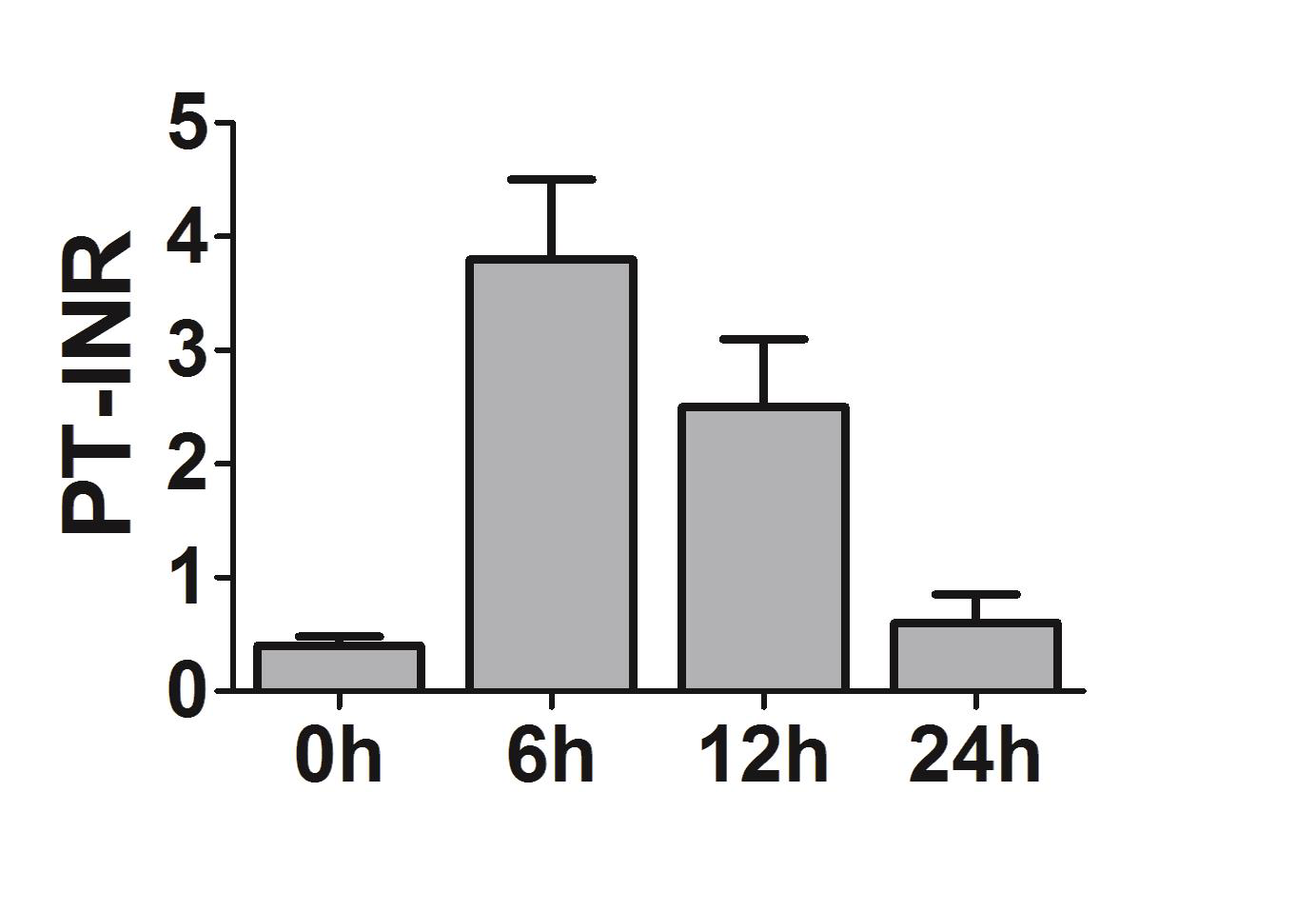

Supplement: Additional file 1: Figure S1. — The PT-INR values after warfarin withdrawal. After warfarin withdrawal, INR values remained stable for the next 6 h and dropped to normal values after 24 h. Data are shown as mean ± SD. [file 12974_2016_661_MOESM1_ESM.tif]

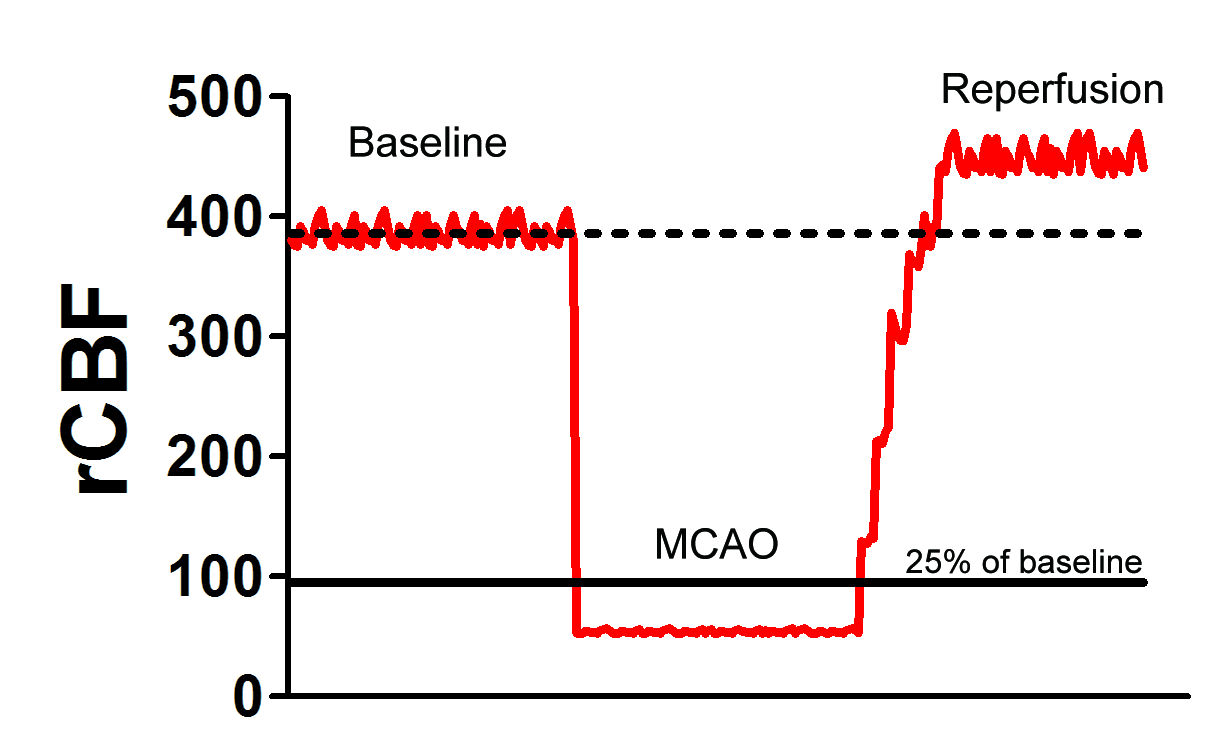

Supplement: Additional file 2: Figure S2. — The rCBF levels in the ischemia and reperfusion stages in MCAO mice. A coated filament was placed on the right middle cerebral artery (MCA) with concurrent recording of laser Doppler cerebral blood flow. In the ischemia stage, the rCBF decreased to <25 % of baseline. After 45 min, the filament was removed and the rCBF increased to 110 % of baseline. [file 12974_2016_661_MOESM2_ESM.tif]

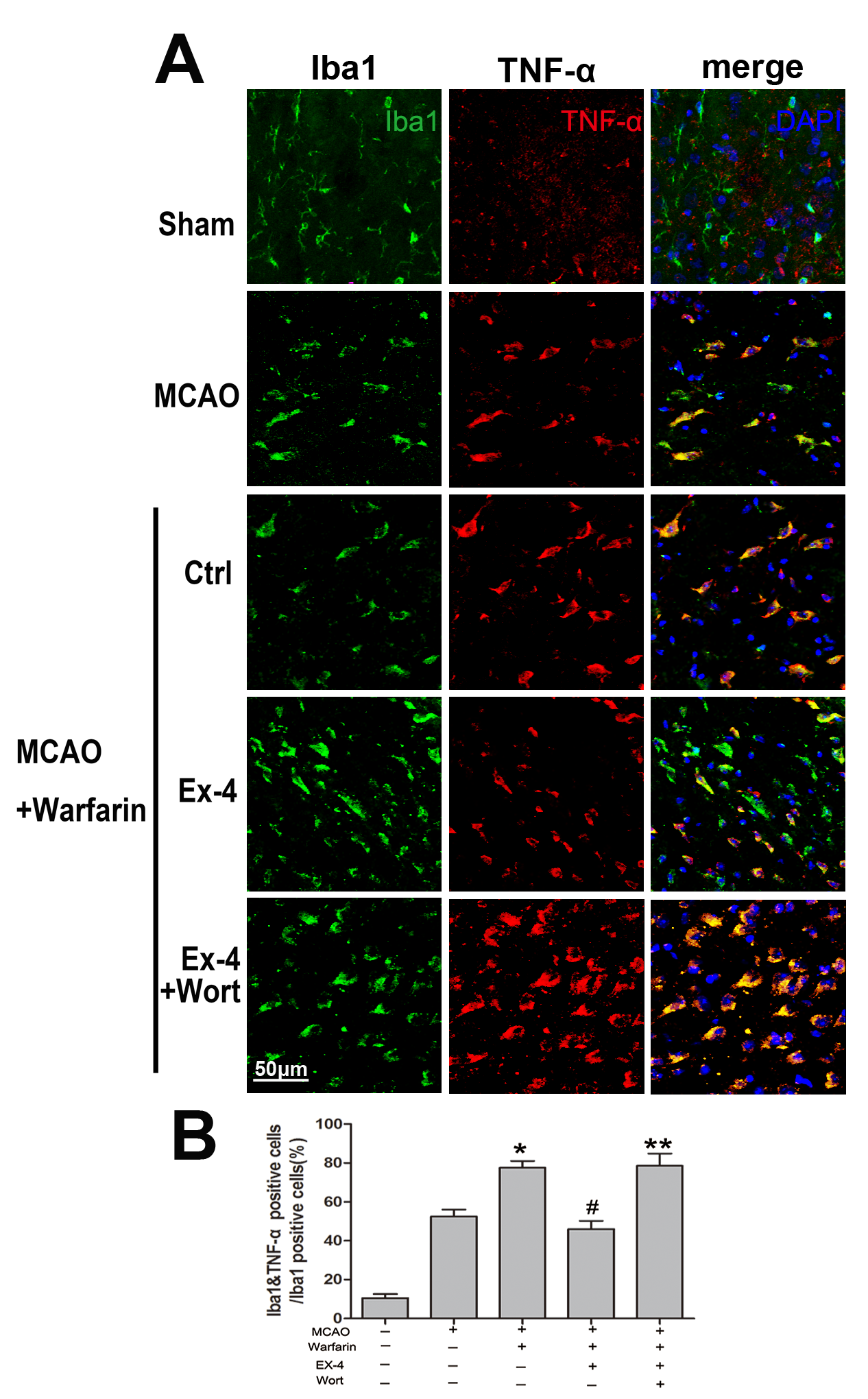

Supplement: Additional file 3: Figure S3. — Representative immunofluorescence images showed co-localization of Iba1 (green) and TNF-α (red) in microglia. Immunostaining of Iba1(green), TNF-α(red), and DAPI (blue) was performed in the cortical and subcortical areas supplied by the middle cerebral artery. (A) Representative immunofluorescence images showed the percentage of Iba1+/TNF-α + cells to total Iba1+ cells was increased after warfarin treatment. EX-4 treatment reduced the Iba1+/TNF-α + cells percentage, whereas wortmannin blocked this effect of EX-4. Scale bar 50 μm. (B) Quantitative analysis of Iba1 and TNF-α double positive cells/Iba1-positive cells. [file 12974_2016_661_MOESM3_ESM.tif]
